# Detection of blood pathogens in camels and their associated ectoparasitic camel biting keds, *Hippobosca camelina*: the potential application of keds in xenodiagnosis of camel haemopathogens

**DOI:** 10.12688/aasopenres.13021.2

**Published:** 2020-05-20

**Authors:** Kevin O. Kidambasi, Daniel K. Masiga, Jandouwe Villinger, Mark Carrington, Joel L. Bargul

**Affiliations:** 1 Department of Biochemistry, Jomo Kenyatta University of Agriculture and Technology, Nairobi, Nairobi, P.O.Box 62000-00200, Kenya; 2 Animal Health Department/Molecular Biology and Bioinformatics Unit, International Centre of Insect Physiology and Ecology, Nairobi, P.O. Box 30772-00100, Kenya; 3 Department of Biochemistry, University of Cambridge, Tennis Court Road, Cambridge, CB2 1QW, UK

**Keywords:** Camelus dromedarius, Hippobosca camelina, haemopathogens, xenodiagnosis, high-resolution melting analysis

## Abstract

**Background: **Major constraints to camel production include pests and diseases. In northern Kenya, little information is available about blood-borne pathogens circulating in one-humped camels ( *Camelus dromedarius*) or their possible transmission by the camel haematophagous ectoparasite, *Hippobosca camelina*, commonly known as camel ked or camel fly. This study aimed to: (i) identify the presence of potentially insect-vectored pathogens in camels and camel keds, and (ii) assess the potential utility of keds for xenodiagnosis of camel pathogens that they may not vector.

**Methods: **In Laisamis, northern Kenya, camel blood samples (n = 249) and camel keds (n = 117) were randomly collected from camels. All samples were screened for trypanosomal and camelpox DNA by PCR, and for *Anaplasma, Ehrlichia, Brucella, Coxiella, Theileria*, and *Babesia* by PCR coupled with high-resolution melting (PCR-HRM) analysis.

**Results: **In camels, we detected *Trypanosoma vivax* (41%), *Trypanosoma evansi* (1.2%), and “ *Candidatus* Anaplasma camelii” (68.67%). In camel keds, we also detected *T. vivax* (45.3%), *T. evansi* (2.56%), *Trypanosoma melophagium* (1/117) (0.4%), and “ *Candidatus* Anaplasma camelii” (16.24 %). Piroplasms ( *Theileria* spp. and *Babesia* spp.), *Coxiella burnetii*, *Brucella* spp., *Ehrlichia* spp., and camel pox were not detected in any samples.

**Conclusions: **This study reveals the presence of epizootic pathogens in camels from northern Kenya. Furthermore, the presence of the same pathogens in camels and in keds collected from sampled camels suggests the potential use of these flies in xenodiagnosis of haemopathogens circulating in camels.

## Introduction

Camels are the most valuable livestock for pastoralist farmers living in arid and semi-arid lands (ASALs) in Kenya ( Mochabo *et al*., 2005). Among other benefits, they provide milk, meat, transport, and income through sale of animal products ( Faye, 2014; Oryan *et al*., 2008). There are no other livestock species that have such versatile uses to pastoralists living in ASALs ( Faye, 2014). Over three million one-humped camels are estimated to be in northern Kenya ( FAOSTAT, 2015; KNBS, 2010), which represents the third largest camel population in Africa after Somalia and Sudan ( Lamuka *et al*., 2017). Camels are resilient to harsh conditions of ASAL regions characterized by long periods of drought, scarcity of vegetation and water, and unpredictable rainfall. However, camel pests and diseases are the major constraints to camel production ( Higgins, 1985; Kassa *et al*., 2011; Mochabo *et al*., 2005). Additionally, the constant association between camels and humans, co-herding of livestock species, and communal watering of animals, as well as sharing of water troughs by the domestic and wild animals, exacerbate the spread of zoonotic diseases, which poses a great risk to public health among livestock and humans in Kenya’s north ( Bengis *et al*., 2002; Kazoora *et al*., 2014; Lamuka *et al*., 2017; Younan & Abdurahman, 2014). Thus, there is a need for constant surveillance of infectious agents circulating within the camel herds in order to guide control and treatment of these diseases.

Camels are vertebrate hosts of various haematophagous arthropods including *Hippobosca* spp. (also known as keds or hippoboscids), horse flies, stable flies, *Lyperosia* spp., and ticks ( Higgins, 1985). In addition to the direct effects such as blood loss, annoyance, and painful feeding bites, these biting pests can be vectors of infectious pathogens ( Baldacchino *et al*., 2013; Higgins, 1985; Young *et al*., 1993). Biting flies such as tabanids and *Stomoxys* have been implicated in the transmission of viruses (including bluetongue and Rift Valley fever viruses), rickettsiae (e.g. *Anaplasma, Coxiella*), *Bacillus anthracis*, and protozoa ( *Besnoitia besnoiti*, *Haemoproteus metchnikovii*, *Trypanosoma theileri*, *Trypanosoma evansi*, *Trypanosoma equiperdum*, *Trypanosoma vivax*, *Trypanosoma congolense*, *Trypanosoma simiae*, *Trypanosoma brucei*) in their specific vertebrate hosts (reviewed by Baldacchino *et al*., 2013).

Hippoboscids (keds) are obligate haematophagous ectoparasites of mammals and birds. They belong to the family Hippoboscidae within the superfamily Hippoboscoidae ( Petersen *et al*., 2007; Rahola *et al*., 2011). This family of haematophagous dipterans is divided into three subfamilies, *Lipopteninae*, *Ornithomyinae*, and *Hippoboscinae* ( Rani *et al*., 2011). *Hippoboscidae* and *Glossinidae* (tsetse; i.e. the definitive vector of African trypanosomes) belong to the same superfamily *Hippoboscoidae*, which is characterized by adenotrophic viviparity ( Petersen *et al*., 2007). Members of *Hippoboscidae* act as vectors of several infectious agents including protozoa, bacteria, helminths, and viruses ( Rahola *et al*., 2011). *Hippobosca camelina* is the predominant ectoparasite of camels in northern Kenya. This haematophagous fly acquires blood meals mainly from camels for its nourishment and reproduction. The role of keds in disease transmission is not well established. Furthermore, as primarily long-term camel blood-feeders, they may have potential in xenosurveillance of pathogens within camel herds that they may not transmit. Therefore, this study was undertaken to (i) detect the presence of infectious viruses, bacteria, protozoa, and rickettsial pathogens, particularly those responsible for zoonoses, in camels and hippoboscids associated with them, and (ii) study the potential utility of hippoboscids in xenodiagnosis.

## Methods

### Study area

**Figure 1.**
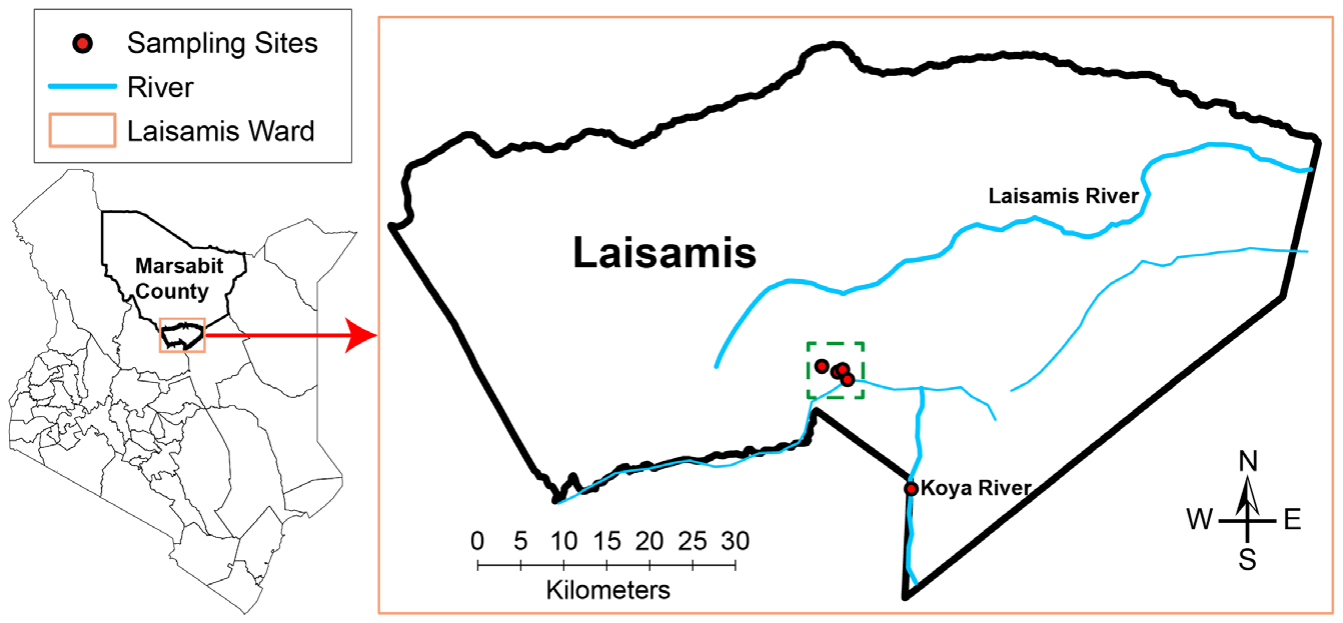
A map of Kenya showing the study sites in Laisamis, Marsabit County, Kenya. Camel blood samples were collected from camel herds during the day, shortly after drinking water from the wells dug along Koya semi-permanent river (circle filled in red), whereas camel keds were collected from the same herds later at night when these camels returned to their temporary settlements shown on the map by circles (filled in red) inside a dotted green square.

The study was carried out in Laisamis (1° 36' 0" N 37° 48' 0" E, 579 m above sea level) located in Marsabit County, northern Kenya ( Figure 1). The County of Marsabit in Kenya has a total area of 70,961km ^2^ and occupies the extreme part of northern Kenya (Source: County Commissioner’s Office, Marsabit, 2013). Area of the Laisamis sub-County that consists of four County Assembly Wards is 20,290 km ^2^ with a population of 84,056 people consisting of about 41,240 males and 42,871 females ( KNBS, 2013). Laisamis electoral ward, one of the four County Assembly Wards of Laisamis sub-County in Marsabit County, has an area of 3,885 km ^2^. A total population of 203,320 camels was reported in Marsabit County, where our present study was conducted ( SSFR, 2017).

### Weather conditions

The average temperature in Laisamis is 26.5°C (19°C – 30°C; March is the warmest month, whereas July is the coldest month of the year). About 413 mm of precipitation falls annually, the average rainfall amounts and rain days differ between years. Long wet seasons occur mostly during April - June, while short wet seasons are experienced in October - December. On the other hand, short dry seasons occur between January and March, whereas long dry spells are experienced between July and September ( SSFR, 2017). However, unpredictable and irregular climatic patterns are becoming more common, with no rainfall in some years leading to frequent droughts in the arid and semi-arid regions of northern Kenya.

### Study design and sample collection

This field study was cross-sectional in design and involved opportunistic sampling of camels from diverse geographical locations as they converge at specific water drinking points. Daily sampling of camels found along the river was convenient strategy considering that camel owners are nomadic pastoralists with busy lifestyles characterized by long distance movements together with their animals and other belongings.

Due lack of historical data on camel diseases in Laisamis sub-County, there was no basis for calculation of the sample sizes, thus we collected as many samples as possible during the sampling duration.

We did not have data on the total number of camel herds kept by the pastoralist community whose main occupation at 87% is livestock herding ( SSFR, 2017). We defined camel herd as a group of camels that spend significant amount of time together by living, feeding, or migrating together. Camels in each herd ranged from 8 – 90 camels.

### Camel blood samples

In September 2017, 249 clinically healthy dromedary camels of both sexes (203 females and 46 males) were sampled in Laisamis sub-County, along Koya River (01° 23’ 11” N, 37° 57' 11.7" E). Koya River was selected as sampling site as it contains permanent watering points. Sampling was preferred in dry season of September when the camel ked densities are highest in contrast to the wet season. We sampled all camels in each and every herd at water drinking points for five consecutive days.

About 5 mL of camel blood was drawn from jugular vein into a heparinised vacutainer and immediately preserved in liquid nitrogen at -196°C for transportation to molecular biology laboratories at the International Centre of Insect Physiology and Ecology ( *icipe,* Nairobi) for analysis.

### Collection of camel keds, *H. camelina*

**Figure 2.**
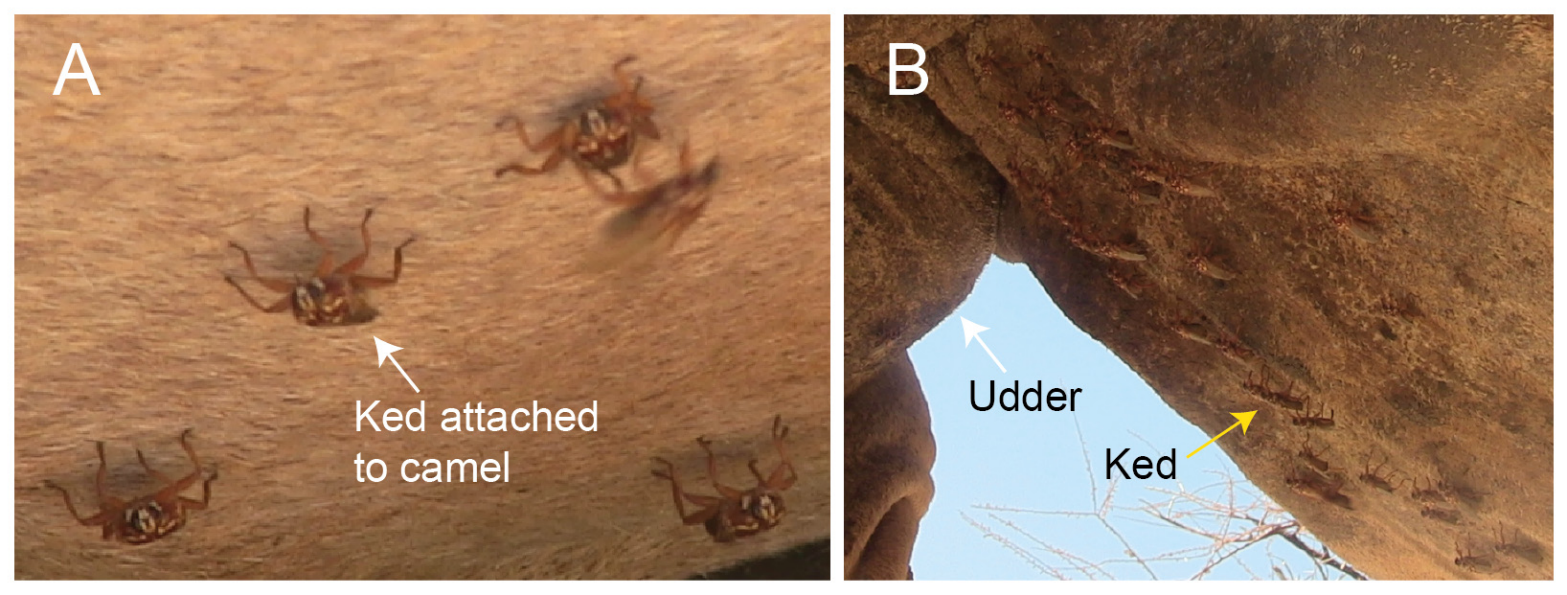
*Hippobosca camelina* flies infesting camels. These ectoparasites are mostly found on the belly of the host as shown in **A** & **B**. Over 30 flies were found concentrated on a small section of underbelly of the camel next to the udder. These flies mostly infest the underbelly and occasionally on the other parts of the camel’s body where they are not prone to disturbance by the host.

Camel keds closely associate and move with their host as they firmly attach to the hairs on camel’s skin using tarsal claws. These blood feeders are mainly observed on the underbelly ( Figure 2), although they can be found on other parts of the body such as the neck and hump. Since we observed that keds are best collected under the cover of darkness at night, we collected blood samples at the water drinking point, then later in the evening followed the same camel herds for fly collection. Flies were collected off camels from four sites (Sarai – 01° 30’ 33.2” N, 037° 52’ 34.4” E; Sarai Maririwa/Kilakir – 01° 35’ 20.5” N, 037° 48’ 39.7” E; Lapikutuk Lelembirikany’ – 01° 30’ 42.9” N, 037° 52’ 53.5” E; Noldirikany’ – 01° 30’ 04.2” N, 037° 54' 50.7" E) by handpicking using spotlights that were briefly switched on and off in order to locate flies on the camels. Camel keds were randomly collected from 21 sampled camel herds in 5 days and we aimed to collect all camel flies found on the camel’s body in all sampled herds. Freshly collected camel keds were preserved in absolute isopropanol and transported to *icipe* for molecular screening of infectious agents. Morphological identification of camel keds was done through comparison with known hippoboscid collections at the Zoology museum of the University of Cambridge (UK), and the Natural History Museum in London. DNA barcoding of COI gene to resolve species of keds was unsuccessful possibly because these flies are little studied and have poor representation in the databases.

### Collection of other biting flies

In order to determine occurrence of tsetse flies and other species of haematophagous biting flies found in Laisamis sub-County and Koya, we deployed monoconical traps, using cow urine and acetone as attractants. Three traps were deployed per site on daily basis from 09:00 - 18:00 next to livestock pens and near watering points along Laisamis River. The inter-trap distance was at least 100 meters. Daily trap collections were pooled, fly species sorted, counted, and then the flies were preserved in 50 mL Falcon tubes half-filled with absolute ethanol for later morphological identification.

### Ethical approval

This study was undertaken in strict adherence to experimental guidelines and procedures approved by the Institutional Animal Care and Use Committee at *icipe* (REF: IACUC/ICIPE/003/2018). All efforts were made to minimize pain and discomfort during sampling. For instance, camel keepers, with whom camels were familiar, were allowed to restrain their camels for sample collection. Samples were collected after receiving informed verbal consent from camel keepers. All camel keepers were neither able to read nor write, thus verbal rather than the written consent was adopted as the pragmatic approach.

### DNA extraction

Each *H. camelina* fly was surface-sterilized with 70% ethanol and allowed to air dry for 10 min on a paper towel on top a clean bench. Individual flies were placed into a clean 1.5 mL centrifuge tubes containing sterile 250 mg of zirconia beads with 2.0 mm diameter (Stratech, UK) and ground in liquid nitrogen in a Mini-Beadbeater-16 (BioSpec, Bartlesville, OK, USA) for 3 min. Genomic DNA was extracted from camel keds and camel blood samples using DNeasy Blood & Tissue Kit (Qiagen, Hilden, Germany) following the manufacturer’s instructions.

### Detection of pathogen DNA

Detection of *Coxiella burnetii*, *Anaplasma* spp., *Ehrlichia* spp., *Brucella* spp., and piroplasms belonging to *Theileria* and *Babesia* genera employed PCR followed by DNA fragment analysis based on high-resolution melting (HRM) analysis ( Šimenc & Potočnik, 2011) in a Rotor-Gene Q thermocycler (Qiagen, German). *Coxiella burnetii* DNA was screened for using primers ( Table 1) targeting the IS1111 gene ( Tokarz *et al*., 2009). *Anaplasma* and *Ehrlichia* species were detected by PCR amplification using genus-specific primers ( Table 1). In detection of *Anaplasma* and *Ehrlichia* species, the PCR-HRM target for 16S rRNA lies within the longer 16S rRNA region, thus the 1000 bp barcode region (amplified by conventional PCR) instead of 16S rRNA HRM products were sequenced. *Babesia* and *Theileria* spp. DNAs were amplified using primers RLB-F1 and RLB-R1 ( Table 1) targeting the hypervariable V4 region of 18S rRNA genes ( Georges *et al*., 2001). The PCRs were carried out in 10 μL reaction volumes, containing 2.0 μL of 5× HOT FIREPol EvaGreen HRM mix (no ROX) (Solis BioDyne, Estonia), 0.5 μL of 10 pmol of each primer, 6.0 μL PCR water and 1.0 μL of template DNA. For *Brucella* spp., the reactions were carried out in 10 μL reaction volumes, containing 2.0 μL of 5× HOT FIREPol EvaGreen HRM mix (no ROX) (Solis BioDyne, Estonia), and 0.5 μL of 10 pmol of each primer of three primers; *Brucella arbutus* forward primer, *B. melitensis* forward primer, and *Brucella* spp. universal reverse primer targeting the IS711 gene ( Probert *et al*., 2004), 5.5 μL PCR water and 1.0 μL of template DNA. PCR amplification was preceded by an initial enzyme activation at 95°C for 15 min, followed by 10 cycles at 94°C for 20 sec, step-down annealing from 63.5°C with decrements of 1°C after each cycle for 25 sec, and primer extension step at 72°C for 30 sec; then 25 cycles of denaturation at 94°C for 25 sec, annealing at 50.5°C for 20 sec, and extension at 72°C for 30 sec followed by a final elongation at 72°C for 7 min. Immediately after PCR, HRM profiles of amplicons were obtained by increasing temperature gradually from 75 to 90°C at 0.1°C/2 sec increments. Changes in fluorescence with time (dF/dT) were plotted against changes in temperature (°C).

**Table 1.** Primers for amplification of target genes for detection of disease-causing camel pathogens. Primers sequences were sent to inqaba biotec™ (Muckleneuk, Pretoria, South Africa) for synthesis.

Primer name	5’to 3’ sequence	Target organism	Target gene	Product size (bp)	References for primers
IS1111F IS1111F	GTA ATA TCC TTG GGC GTT GAC G ATC TAC GCA TTT CAC CGC TAC AC	*C. burnetii*	*Coxiella* 16S rRNA	242	( Doosti *et al.,* 2014)
*Anaplasma*JV *F* *Anaplasma*JV *R*	CGGTGGAGCATGTGGTTTAATTC CGRCGTTGCAACCTATTGTAGTC	*Anaplasma* spp.	*Anaplasma* 16S rRNA	300	( Mwamuye *et al.,* 2017)
*Ehrlichia*JV F *Ehrlichia*JV R	GCAACCCTCATCCTTAGTTACCA TGTTACGACTTCACCCTAGTCAC	*Ehrlichia* spp.	*Ehrlichia* 16S rRNA	300	( Mwamuye *et al.,* 2017)
EHR16SD 1492R	GGTACCYACAGAAGAAGTCC GGTTACCTTGTTACGACTT	*Ehrlichia & Anaplasma* spp.	16S rRNA	1000	( Parola *et al.,* 2000; Reysenbach *et al.,* 1992)
RLB F RLB R	GAGGTAGTGACAAGAAATAACAATA TCTTCGATCCCCTAACTTTC	*Theileria &* *Babesia* spp.	*Theileria &* *Babesia* 18S rRNA	450	
CMLVC18LF CMLVC18LR	GCGTTAACGCGACGTCGTG GATCGGAGATATCATACTTTACTTTAG	Camel pox virus	C18L gene	243	( Balamurugan *et al.,* 2009)
*B. arbutus* F *B. melitensis* F *Brucella* IS711T R	GCGCTCAGGCTGCCGACGCAA GCGGCTTTTCTATCACGGTATTC GGGTAAAGCGTCGCCAGAAG	*Brucella* spp	IS711		( Probert *et al.,* 2004)
ITS1_CF ITS1_BR	CCGGAAGTTCACCGATATTG TTGCTGCGTTCTTCAAC- GAA	*Trypanosoma* spp.	ITS1	250-720	( Njiru *et al.,* 2005)
ILO 7957F ILO 8091R	GCCACCACGGCGAAAGAC TAATCAGTGTGGTGTGC	*T. evansi*	RoTat 1.2	488	( Urakawa *et al.,* 2001)

Screening of pathogenic animal African trypanosomes and camelpox virus was done by PCR in a ProFlex thermocycler (Applied Biosystems). Trypanosome DNA was amplified by targeting trypanosomal internal transcribed spacer region using the following universal primer sets described by Njiru *et al*. (2005); ITS1_CF and ITS1_BR ( Table 1) in 10 µL PCR volumes containing 0.1 units of Phusion DNA polymerase (Finnzymes, Espoo, Finland), 2 µL of 5× HF buffer, 0.2 µL of 10 mM dNTPs, 0.2 µL of 10 mM of each primer and 6.3 µL of nuclease free water. The PCR conditions were as follows: 98°C for 1 min, 40 cycles of 98°C for 30 sec, 61°C for 30 sec, and 72°C for 45 sec, with a final elongation step of 7 min at 72°C. Camelpox virus C18L gene was amplified using CMLV C18LF and CMLV C18LR primers described by Balamurugan *et al.* (2009). The PCRs were carried out in a 10-µL reaction mixtures containing 5.0 µL of DreamTaq Green PCR master mix (2×) (Thermo Scientific), 0.5 µL of 10 mM of each primer, 1.0 µL of DNA template, and 3.0 µL of nuclease-free water. The PCR thermocycling conditions included; initial denaturation at 95°C for 3 min, 35 cycles of 95°C for 30 sec, 58°C for 30 sec, 72°C for 30 sec followed with a final elongation of 72°C for 5 min. The PCR amplicons were electrophoresed on 1.5% ethidium bromide-stained agarose gel and visualized under ultraviolet light.

### DNA purification and sequencing

Representative positive samples producing distinct amplicons with expected band sizes relative to the known positive DNA controls were selected for amplification in larger PCR reaction volumes (30-μL). The PCR amplicons were separated by electrophoresis in ethidium bromide-stained 1.5% agarose gels and visualized under ultraviolet light. The target bands were excised and gel purified using QIAquick PCR purification kit (Qiagen, Germany) according to manufacturer’s instructions. The purified amplicons were sent to Macrogen Inc. (Netherlands) for Sanger sequencing.

Since it is not possible to resolve the trypanozoon species using ITS1 primers, which give 480-bp PCR product sizes ( Njiru *et al*., 2005), two samples positive for *Trypanozoon*, one from camel and the other from hippoboscid, were amplified using ILO 7957F and ILO 8091R primers ( Table 1) targeting RoTat 1.2 VSG gene described by Urakawa *et al.* (2001).

To identify the *Anaplasma* species associated with the HRM peaks observed, amplicons of two samples positive for *Anaplasma* spp., one from camels and one from camel ked, were selected for sequencing using AnaplasmaJVF and *Anaplasma*JVR targeting 300-bp of *Anaplasma* 16S rRNA genes. These primers could not resolve *Anaplasma* to species level. To resolve the *Anaplasma* to species level, a longer 1000-bp fragment of *Anaplasmataceae* 16S rRNA gene was further amplified by conventional PCR using published primers EHR16SD and 1492R ( Parola *et al*., 2000; Reysenbach *et al*., 1992, Table 1), and sequenced. The PCR amplifications were performed in a ProFlex PCR system (Applied Biosystems by life technologies) with the following cycling conditions: 95°C for 15 min; two cycles of 95°C for 20 sec, 58°C for 40 sec, and 72°C for 90 sec; three cycles of 95°C for 20 sec, 57°C for 30 sec, 35 cycles of 95°C for 20 sec, 56°C for 40 sec and 72°C for 90 sec, and a final extension at 72°C for 10 min ( Bastos *et al*., 2015).

Sequences obtained in the study were deposited in GenBank database with the following accession numbers: short 16S rRNA of *Anaplasma* spp. in camel (MN306317) and camel ked (MN306316); full length 16S rRNA of “ *Candidatus* Anaplasma camelii” (MN306315), *T. vivax* ITS1 in camel (MK880188), and camel ked (MK880189); RotTat 1.2 VSG gene of *T. evansi* in camel (MK867833) and camel ked (MK867832).

### Data analysis

Data on sampled camels and hippoboscids were entered into Microsoft Excel spreadsheet, version 12.3.1. Georeferenced data of the sampling sites and administrative boundaries data from Kenya Open Data in shapefile data format, were loaded into the ArcMap component of ArcGIS 10.6 software. The component was then used to design and generate the map layout of the sampling sites.

Using the MAFFT plugin in Geneious Prime 2019.1.1 software version (created by Biomatters) ( Kearse *et al*., 2012), all study nucleotide sequences were edited and aligned with related sequences identified by querying in the GenBank nr database using the Basic Local Alignment Search Tool ( www.ncbi.nlm.nih.gov/BLAST/).

## Results

### Detections of pathogens

Out of 249 camel samples screened, 102 (40.96%) tested positive for trypanosomes by ITS1 PCR ( Table 2). All trypanosome positive samples were infected with *T. vivax* showing an expected band of 250 bp and confirmed by amplicon sequencing. Mixed infections with *T. vivax* (250-bp band) and *T. evansi* (480-bp band) were detected in three camels (1.2%).

**Table 2.** Summary of selected pathogens detected in camels and *Hippobosca camelina.*

Pathogen	Prevalence in camels ( *n* = 249)	Prevalence in *H. camelina* ( *n* = 117)
*Trypanosoma vivax*	102 (41%)	53 (45.3%)
*Trypanosoma evansi*	3 (1.2%)	3 (2.56%)
*Trypanosoma melophagium*	0 (0%)	1 (0.85%)
“ *Candidatus* Anaplasma camelii”	171 (68.67%)	19 (16.24%)

**Figure 3.**
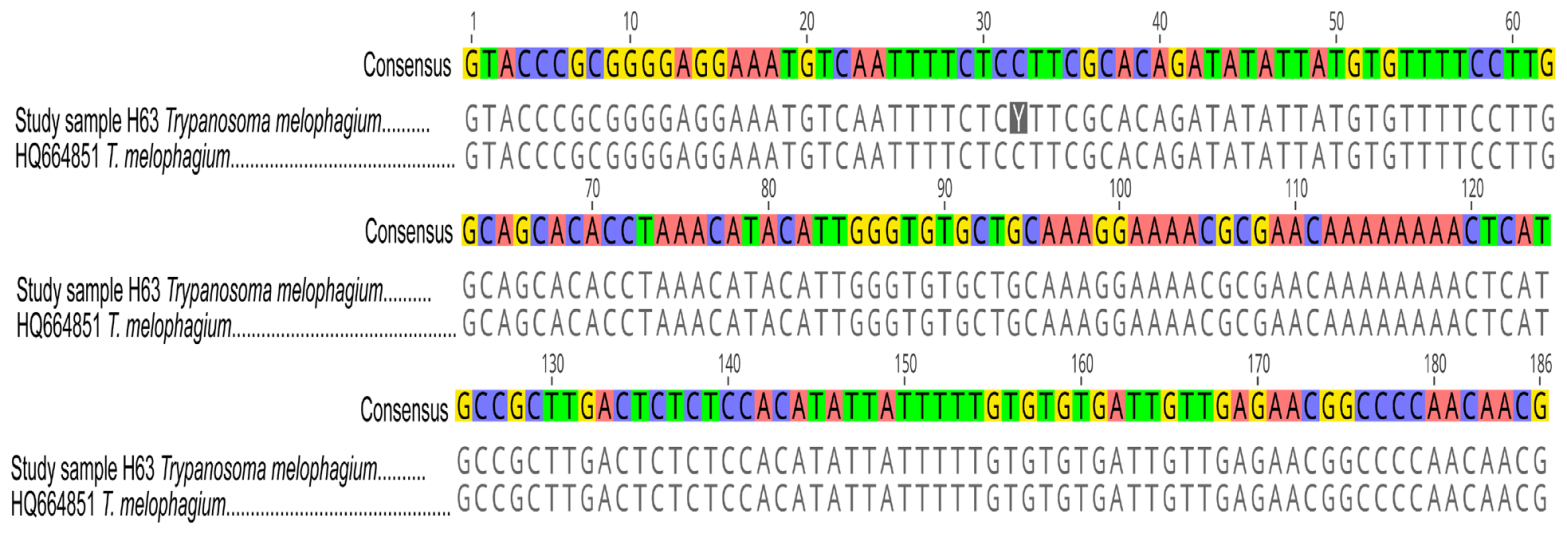
Pairwise alignment of ITS1 sequences of *T. melophagium*. ITS1 sequence of *T. melophagium* in study sample H63 was aligned with highly identical sequence (HQ664851) from GenBank. At position 32, there is a nucleotide change from C in the sequence from GenBank to Y in the sequence from this study.

Out of 117 *H. camelina* samples, 53 (45.30%) were infected with trypanosomes, all of which had *T. vivax*. Three flies (2.56%) had mixed infections with *T. evansi* and *T. vivax*. Additionally, one fly had double infection of *T. vivax* and *Trypanosoma* sp. amplicon of about 400 bp. The 400 bp *Trypanosoma* sp. was sequenced using the ITS1 marker and shared 98.14% identity with *Trypanosoma melophagium* (GenBank accession HQ664851) that was sequenced from *Melophagus ovinus*, sheep ked, in Croatia. Figure 3 shows pairwise alignment of *T. melophagium* sequence from this study and that from GenBank.

In total 98% of all monoconical trap catches were *Stomoxys calcitrans*, with the remaining 2% consisting of Tabanidae and *H. camelina* ( Table 3). None of the traps caught tsetse (0%) in all sampling locations.

**Table 3.** Biting flies were trapped using monoconical traps with cow urine and acetone as attractants. Daily trap collections were pooled, fly species sorted, counted, and preserved in absolute ethanol for transportation to the Nairobi-based laboratories at icipe. Majority of the fly collections comprised of *Stomoxys calcitrans*. Tsetse flies (genus *Glossina*) were absent in all traps.

Sampling site	Day	*Daily fly captures	Sex (M = male; F = female)
**Kula pesa** 01° 35’ 44.9” N, 037° 48’ 35.8” E	**Day 1**	**12**	12 F – *Stomoxys calcitrans*
	**Day 2**	**8**	5 F – *S. calcitrans*; 1 M & 2 F – *Hippobosca camelina*
	**Day 3**	**4**	3 F – *S. calcitrans*; 1 F – *H. camelina*
	**Day 4**	**12**	9 F & 1 M - *S. calcitrans;* 1 F – *Tabanus* spp.; 1 M – *H. camelina*
	**Day 5**	**5**	4 F – *S. calcitrans*; 1 F – *H. camelina*
	**Day 6**	**11**	1 M & 9 F – *S. calcitrans*; 1 M – *H. camelina*
	**Day 7**	**8**	6 M & 2 F – *S. calcitrans*
**Soweto** 01° 35’ 43.1” N, 037° 48’ 35.7” E	**Day 1**	**2**	2 F – *S. calcitrans*
	**Day 2**	**9**	2 M & 7 F – *S. calcitrans*
	**Day 3**	**7**	1 M & 6 F – *S. calcitrans*
	**Day 4**	**7**	2 M & 4 F – *S. calcitrans*; 1 F – *H. camelina*
	**Day 5**	**2**	2 F – *S. calcitrans*
	**Day 6**	**9**	9 F – *S. calcitrans*
	**Day 7**	**0**	Biting flies count = 0 (Only house flies were trapped)
**Naigero** 01° 35’ 49.7” N, 037° 49’ 58.1” E	**Day 1**	**33**	8 M & 25 F – *S. calcitrans*
	**Day 2**	**14**	2 M & 12 F – *S. calcitrans*

*Daily fly captures: represent pools of three trap catches per site per day.

**Figure 4.**
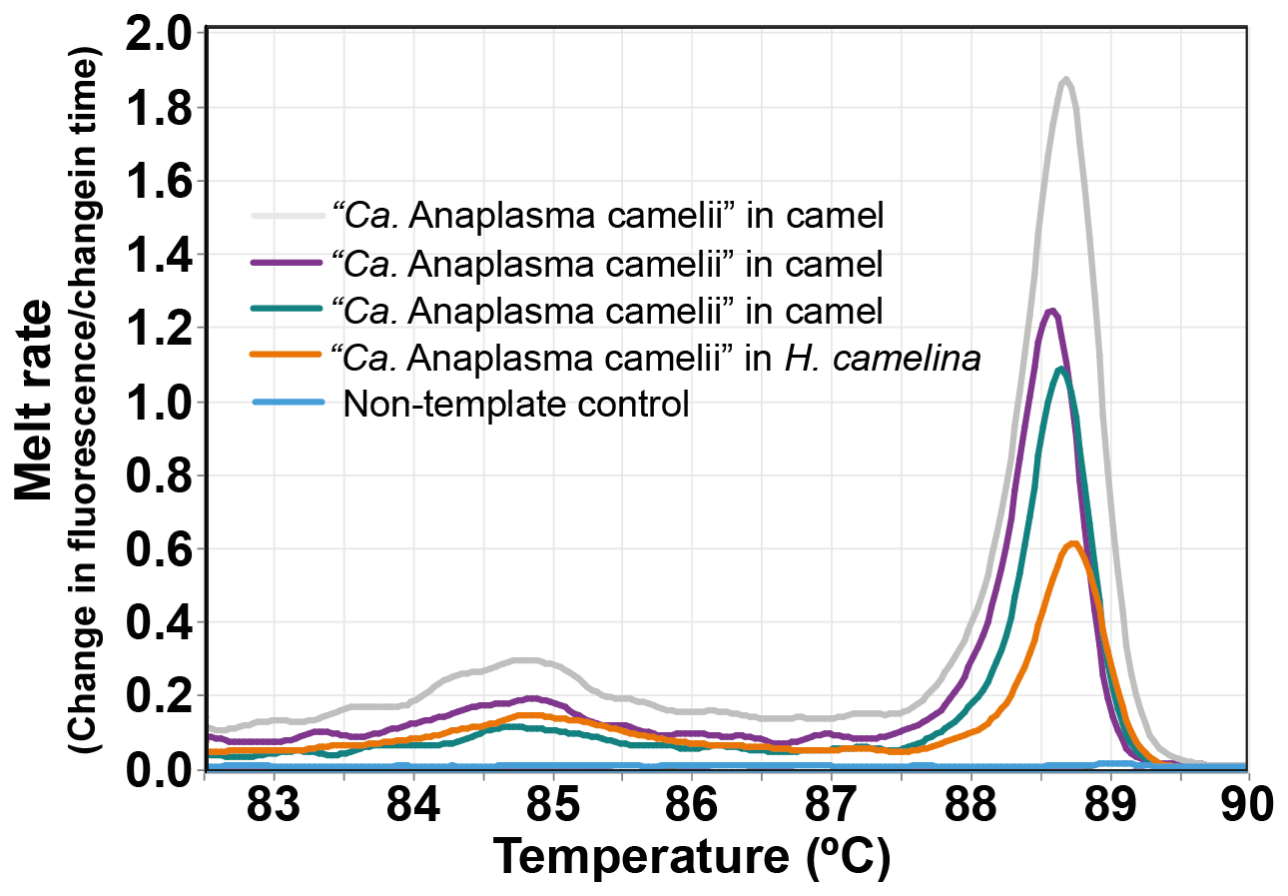
Melt curves of amplification of 16S rRNA of “ *Candidatus* Anaplasma camelii” in camels and camel keds.

**Figure 5.**
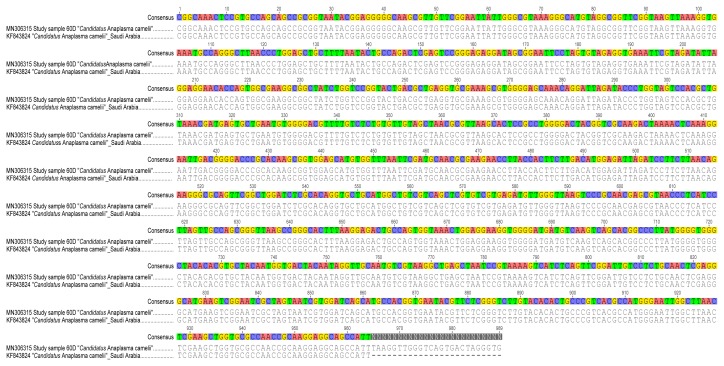
Pairwise alignment of 16S rRNA sequences of “ *Candidatus* Anaplasma Camelii” amplified from camel blood sample 60D with GenBank-retrieved nucleotide sequence (KF843824) showed 100% identity. N is ambiguous nucleotide.

*“Candidatus* Anaplasma camelii” was detected in 68.67% ( *n* = 171/249) of dromedary camels and 16.24% ( *n* = 19/117) of *H. camelina* ( Figure 4). Though the 300-bp *Anaplasma* 16S rRNA sequences could not resolve the *Anaplasma* spp. to species level, analysis of the 1000-bp 16S rRNA nucleotide sequence showed 100% identity with “ *Candidatus* Anaplasma camelii” sequenced from camels in Saudi Arabia (GenBank accession numbers KF843824-KF843825) and Iran (GenBank accession KX765882) ( Figure 5). Piroplasms ( *Theileria* spp. and *Babesia* spp.), *C. burnetii*, *Ehrlichia* spp., *Brucella* spp., and camel pox were not detected either in camels or keds collected from them.

## Discussion

We report the occurrence of similar blood-borne pathogens in dromedary camels and in *H. camelina* flies collected from the same herds. The high infection rates of pathogens in camels ( *T. vivax* = 41%, *T. evansi* = 1.2%, and *Anaplasma* spp. = 68.67%) and flies ( *T. vivax* = 45.3%, *T. evansi* = 2.56%, and *Anaplasma* spp. = 16.24%) suggest the potential of these camel biting flies in disease transmission as well in diagnosis of haemopathogens found in camels. Thus, our findings show *T. vivax* as the most predominant species causing trypanosomiasis in camels sampled in September 2017 from Koya and its surroundings. Similarly, we recorded high fly infection rates of 45.3% caused by the same parasite, *T. vivax.* These high *T. vivax* infection rates could be attributed to mechanical transmission by several biting flies such as *Tabanus* spp. and *Stomoxys* species ( Baldacchino *et al*., 2013) that were collected in this study using monoconical traps ( Table 3). We hypothesize that camels are initially infected with trypanosomes when nomadic pastoralists occasionally move their livestock into distant neighbouring tsetse-infested regions in search of pasture and water, and thereafter maintenance of pathogen transmission among camels continues throughout the year via mechanical transmission in the process of bloodmeal acquisition by biting flies such as camel hippoboscids.

Tsetse flies were not caught despite repeated attempts to trap them in major sampling sites using monoconical traps with cow urine and acetone ( Table 3). This ASAL region in Marsabit south is generally arid, hot, and dry (low humidity) with poor vegetation cover that presumably renders it uninhabitable for tsetse flies. However, by using robust landscape and climatic data modeling, Marsabit has generally been predicted as a region with potential risk of tsetse infestation ( Moore & Messina, 2010).

Currently, little is known about the prevalence and transmission of vector-borne diseases of livestock in northern Kenya, mostly because these animals belong to the marginalized poor nomadic pastoralists whose economic welfare is neglected. Thus, information about *T. vivax* in Kenyan camels is scarce. However, *T. vivax* has been reported in camels in Sudan, Ethiopia, and Nigeria ( Fikru *et al*., 2015; Mbaya *et al*., 2006; Mossaad *et al*., 2017). The pathogenicity of *T. vivax* infection in camels is not well understood, but it is known to be pathogenic to cattle, sheep, equines, and goats ( Galiza *et al*., 2011). Our finding of a high trypanosome infection rate (40.96%) in camels, consisting predominantly of *T. vivax*, suggests that infected camels could act as parasite reservoirs for other susceptible and often co-herded livestock species in the region.

*Trypanosoma evansi* infection rates of 1.2% in camels reported in this study is much lower than earlier reports of up to 46% infections in other regions of Kenya ( Ngaira *et al*., 2004; Njiru *et al*., 2004). These regional variations in prevalence of *T. evansi* infection could result from seasonal disease outbreaks, variations in the micro-climatic conditions, disease stability in endemic zones, and the presence of competent insect-vectors, among other factors, including differences in the study designs and time lapse. Our study design has key limitations in comparative studies because sampling was not done during wet season, thus the relationship between seasonality versus prevalence of haemoparasites and vector density could not be established. *Trypanosoma evansi* is considered the most important protozoan pathogen of dromedary camels in Kenya ( Njiru *et al*., 2004) and has been reported to infect horses and donkeys in other parts of the world ( Desquesnes *et al*., 2013). Additionally, cases of atypical *T. evansi* infections have been reported in humans (reviewed in Truc *et al*., 2013) and was specifically attributed to frameshift mutations of apolipoprotein L-I in one of the patients ( Vanhollebeke *et al*., 2006), thus resulting into lack of immunity against African animal trypanosomes.

“ *Candidatus* Anaplasma camelii” infection, which we report here for the first time in Kenyan camels, was the most prevalent (68.67%) amongst all detected pathogens in this study. This emergent *Anaplasma* pathogen was recently detected in 35.85% of Moroccan dromedary camels ( Ait Lbacha *et al*., 2017). *Anaplasma* sp. isolated from Kenyan camels was 100% identical to the GenBank-retrieved “ *Candidatus* Anaplasma Camelii” 16S rRNA nucleotide sequences from Saudi Arabia and Iran dromedary camels, hence suggesting a common origin of this pathogen ( Figure 5). High prevalence of camel anaplasmosis could be attributed to ticks (the definitive vector of *Anaplasma*) that were present on 100% of camels, and in addition, biting flies such as *Stomoxys calcitrans* promote mechanical transmission ( Scoles *et al*., 2005). Although previous studies could not prove the ability of cattle keds ( *Hippobosca rufipes*) to transmit *Anaplasma marginale* ( Potgieter *et al*., 1981), it is possible that in the process of bloodmeal acquisition, camel keds, *Hippobosca camelina,* could mechanically transmit anaplasmosis via contaminated mouthparts (unpublished study; authors from the present study). The clinical role of “ *Candidatus* Anaplasma camelii” in camels is uncertain, but oedema has been observed in infected camels ( Ait Lbacha *et al*., 2017).

The high prevalence of “ *Candidatus* Anaplasma camelii” in healthy dromedary camels indicates the possible role of camels as reservoir hosts for maintaining its circulation. Further research is needed to determine the zoonotic potential of this tick-borne pathogen. This is important because cases of human infection with *Anaplasma platys* and *Ehrlichia canis,* that are closely related to the emergent “ *Candidatus* Anaplasma camelii” pathogen, have been reported ( Arraga-Alvarado *et al*., 2014; Doudier *et al*., 2010). This possibly zoonotic pathogen of camels ( Lbacha *et al*., 2017) should stimulate the need for increased surveillance by veterinary and public health partners to mitigate spread of infection to humans and other animals.

We detected *T. vivax*, *T. evansi*, *T. melophagium*, and *Anaplasma* species in *Hippobosca camelina*. Detection of identical haemopathogens in *H. camelina* flies as well as in camels from which they were collected suggests that this fly could play role in the transmission of infectious agents amongst its bloodmeal hosts. The ability of *H. camelina* as efficient flier facilitates fast movements on the host or between camel hosts, hence increasing its chances of acquiring infected bloodmeal that could transmitted to the next host following interrupted feeding. Various hippoboscid species have been implicated in transmission of pathogens ( Rahola *et al*., 2011). For instance, *Hippobosca longipennis* is thought to transmit the larva of filarial nematode *Acanthocheilonema dracunculoides* to hyenas and domestic dogs ( Nelson, 1963; Rani *et al*., 2011). Louse flies, *Melophagus ovinus,* play a role in the transmission of *Bartonella* spp. among ruminants ( Halos *et al*., 2004). Another louse fly known as *Icosta americana* is suspected to transmit West Nile virus in North America ( Farajollahi *et al*., 2005). Further studies are needed to determine the vectorial competence of *H. camelina* in the transmission of pathogens.

### Potential role of *H. camelina* in xenodiagnosis

Our findings consistently show that the blood-borne pathogens detected in camels are also present in *H. camelina* collected from them (i.e. sampled camels). It is likely that when keds bite camel hosts to acquire bloodmeals, they also take up haemopathogens if the camel is infected.

*H. camelina* acquires bloodmeals from camels for nutrition and reproduction. Adult stage of keds are obligate blood-feeding ectoparasites of camels that hardly leave their host, unless disturbed and even then, they quickly find the next host. Keds have claspers for firm attachment to the skin hairs of the host during feeding or resting. These flies that prefer to always remain on the vertebrate host, preferentially attach to specific body parts, commonly on the underbelly ( Figure 2) of the camel, near or on the udder, or the perineal region where they are not easily disturbed during bloodmeal acquisition ( Higgins, 1985). These features of camel keds make them good candidates for xenosurveillance and they can be collected easily for molecular screening to detect pathogens acquired from naturally infected camels in the process of feeding. Screening of camel keds for indirect detection of pathogens present in camels, from which they were collected, will save on time and cost. Collection of keds off camels was much easier and required relatively less time than blood sampling. We employed six field assistants to restrain each camel for blood collection, veterinary personnel who collected blood samples, and additional three assistants to carry cool boxes and consumables, ensure accurate labeling of samples and storage, and recording of baseline data. On the other hand, only about four field assistants were needed to collect keds from camel herds, resulting in >50% reduction in labour costs and the required human resource. Fly collection also took shorter time as it was not necessary to restrain camels. Importantly, this xenosurveillance detection provides a less invasive approach than the currently available painful blood collection procedures that pose huge risk to the handlers as camels could occasionally cause severe and even fatal injuries through bites ( Abu-Zidan *et al*., 2012) or by kicking with their legs. In a similar indirect pathogen detection approach, previous reports showed the utility of mosquitoes in xenosurveillance of human pathogens ( Grubaugh *et al*., 2015).

Additionally, a novel *Trypanosoma* sp. closely related to *Trypanosoma melophagium* was detected in one camel ked, *H. camelina* (1/117) *,* but not in camels. This host-specific parasite of sheep, called *T. melophagium*, has never been reported to cause camel infections. Interestingly, *T. melophagium* is known to be solely transmitted by wingless sheep ked called *Melophagus ovinus* ( Gibson *et al*., 2010). We conducted a survey of sheep keds among small ruminants in our study area in northern Kenya and found that they are absent in the region (unpublished study; authors from the present study). Thus, molecular detection of *T. melophagium* in a single camel-specific ked that was collected from camel raises an interesting question about the origin of this parasite. *H. camelina* acquired contamination possibly from *T. melophagium*-infected vertebrate host through bloodmeal. Further studies are needed to determine the vectorial competence of *H. camelina* in transmission of *T. melophagium*.

## Conclusions

Our findings suggest the potential role of *H. camelina* in xenodiagnosis for detection of haemopathogens in camels, thus bypassing the need to obtain blood samples via jugular venipuncture for pathogen detection. Further studies to profile additional blood-borne pathogens including viral diseases occurring both in camels and *H. camelina* that fed on them, will be crucial for supporting usage of hippoboscids in xenomonitoring of camel diseases.

## Data availability

### Underlying data

16S r RNA of *Anaplasma* sp. in camel, Accession number MN306316: https://www.ncbi.nlm.nih.gov/nuccore/MN306316

16S r RNA of *Anaplasma* sp. in camel ked, Accession number MN306317: https://www.ncbi.nlm.nih.gov/nuccore/MN306317

16S rRNA of *Candidatus* Anaplasma camelii, Accession number MN306315: https://www.ncbi.nlm.nih.gov/nuccore/MN306315

*T. vivax* ITS1 in camel, Accession number MK880188: https://www.ncbi.nlm.nih.gov/nuccore/MK880188

*T. vivax* ITS1 in camel ked, Accession number MK880189: https://www.ncbi.nlm.nih.gov/nuccore/MK880189

RotTat 1.2 VSG gene of *T. evansi* in camel, Accession number MK867833: https://www.ncbi.nlm.nih.gov/nuccore/MK867833

RotTat 1.2 VSG gene of *T. evansi* camel ked, Accession number MK867832: https://www.ncbi.nlm.nih.gov/nuccore/MK867832

Figshare: Detection of Anaplasma and Trypanosomes in camels and camel keds, https://doi.org/10.6084/m9.figshare.10050587 ( Kidambasi *et al*., 2019).

This project contains the following underlying data:

- Raw HRM Rotor-Gene Q data files of *Anaplasma* spp. amplification in camels and camel keds. HRM data files can be accessed using Rotor-gene Q software.
- Gel visualization images of resolved PCR amplicons for detection of African trypanosomes in camels and camel keds.

Data are available under the terms of the Creative Commons Zero "No rights reserved" data waiver (CC0 1.0 Public domain dedication).

## References

[ref-1] Abu-Zidan FM Eid HO Hefny AF: Camel bite injuries in United Arab Emirates: a 6 year prospective study. *Injury.* 2012;43(9):1617–1620. 10.1016/j.injury.2011.10.039 22186231

[ref-2] Ait Lbacha H Zouagui Z Alali S: “ *Candidatus* anaplasma camelii” in one-humped camels ( *Camelus dromedarius*) in Morocco: a novel and emerging *anaplasma* species? *Infect Dis Poverty.* 2017;6:1. 10.1186/s40249-016-0216-8 28160773 PMC5292149

[ref-3] Arraga-Alvarado CM Qurollo BA Parra OC: Case report: Molecular evidence of *Anaplasma platys* infection in two women from Venezuela. *A J Trop Med Hyg.* 2014;91(6):1161–1165. 10.4269/ajtmh.14-0372 25266347 PMC4257640

[ref-4] Balamurugan V Bhanuprakash V Hosamani M: A polymerase chain reaction strategy for the diagnosis of camelpox. *J Vet Diagn Invest.* 2009;21(2):231–237. 10.1177/104063870902100209 19286503

[ref-5] Baldacchino F Muenworn V Desquesnes M: Transmission of pathogens by *Stomoxys* flies (Diptera, Muscidae): a review. *Parasite.* 2013;20:26, 13. 10.1051/parasite/2013026 23985165 PMC3756335

[ref-6] Bastos AD Mohammed OB Bennett NC: Molecular detection of novel *Anaplasmataceae* closely related to *Anaplasma platys* and *Ehrlichia canis* in the dromedary camel ( *Camelus dromedarius*). *Vet Microbiol.* 2015;179(3–4):310–4. 10.1016/j.vetmic.2015.06.001 26096752

[ref-7] Bengis RG Kock RA Fischer J: Infectious animal diseases: the wildlife/livestock interface. *Rev Sci Tech.* 2002;21(1):53–65. 10.20506/rst.21.1.1322 11974630

[ref-8] Desquesnes M Dargantes A Lai DH: *Trypanosoma evansi* and surra: a review and perspectives on transmission, epidemiology and control, impact, and zoonotic aspects. *Biomed Res Int.* 2013;2013:321237. 10.1155/2013/321237 24151595 PMC3789323

[ref-9] Doosti A Arshi A Sadeghi M: Investigation of *Coxiella burnetii* in Iranian camels. *Comp Clin Path.* 2014;23(1):43–46. 10.1007/s00580-012-1567-6

[ref-10] Doudier B Olano J Parola P: Factors contributing to emergence of *Ehrlichia* and *Anaplasma* spp. as human pathogens. *Vet Parasitol.* 2010;167(2–4):149–154. 10.1016/j.vetpar.2009.09.016 19836890

[ref-11] FAOSTAT: Food and Agriculture Organization statistical database.2015 Reference Source

[ref-12] Farajollahi A Crans WJ Nickerson D: Detection of West Nile virus RNA from the louse fly *Icosta americana* (Diptera: Hippoboscidae). *J Am Mosq Control Assoc.* 2005;21(4):474–476. 10.2987/8756-971X(2006)21[474:DOWNVR]2.0.CO;2 16506578

[ref-13] Faye B: The Camel Today: Assets and Potentials. *Anthropozoologica.* 2014;49(2):167–176. 10.5252/az2014n2a01

[ref-14] Fikru R Andualem Y Getachew T: Trypanosome infection in dromedary camels in Eastern Ethiopia: Prevalence, relative performance of diagnostic tools and host related risk factors. *Vet Parasitol.* 2015;211(3–4):175–181. 10.1016/j.vetpar.2015.04.008 26071981

[ref-15] Galiza GJ Garcia HA Assis AC: High mortality and lesions of the central nervous system in trypanosomosis by *Trypanosoma vivax* in Brazilian hair sheep. *Vet Parasitol.* 2011;182(2–4):359–363. 10.1016/j.vetpar.2011.05.016 21664764

[ref-16] Georges K Loria GR Riili S: Detection of haemoparasites in cattle by reverse line blot hybridisation with a note on the distribution of ticks in Sicily. *Vet Parasitol.* 2001;99(4):273–286. 10.1016/s0304-4017(01)00488-5 11511414

[ref-17] Gibson W Pilkington JG Pemberton JM: *Trypanosoma melophagium* from the sheep ked *Melophagus ovinus* on the island of St Kilda. *Parasitology.* 2010;137(12):1799–804. 10.1017/S0031182010000752 20546642

[ref-18] Grubaugh ND Sharma S Krajacich BJ: Xenosurveillance: a novel mosquito-based approach for examining the human-pathogen landscape. *PLoS Negl Trop Dis.* 2015;9(3):e0003628. 10.1371/journal.pntd.0003628 25775236 PMC4361501

[ref-19] Halos L Jamal T Maillard R: Role of Hippoboscidae flies as potential vectors of *Bartonella* spp. infecting wild and domestic ruminants. *Appl Environ Microbiol.* 2004;70(10):6302–6305. 10.1128/AEM.70.10.6302-6305.2004 15466580 PMC522062

[ref-20] Higgins AJ: Common ectoparasites of the camel and their control. *Br Vet J.* 1985;141(2):197–216. 10.1016/0007-1935(85)90153-8 3888345

[ref-21] Kassa T Eguale T Chak H: Prevalence of camel trypanosomosis and its vectors in Fentale district, South East Shoa Zone, Ethiopia. *Veterinarski Arhiv.* 2011;81(5):611–621. Reference Source

[ref-22] Kazoora HB Majalija S Kiwanuka N: Prevalence of *Mycobacterium bovis* skin positivity and associated risk factors in cattle from western Uganda. *Trop Anim Health Prod.* 2014;46(8):1383–1390. 10.1007/s11250-014-0650-1 25187023

[ref-23] Kearse M Moir R Wilson A: Geneious Basic: an integrated and extendable desktop software platform for the organization and analysis of sequence data. *Bioinformatics.* 2012;28(12):1647–1649. 10.1093/bioinformatics/bts199 22543367 PMC3371832

[ref-24] Kidambasi K Masiga DK Villinger J: Detection of Anaplasma and Trypanosomes in camels and camel keds. * figshare.*Dataset.2019 10.6084/m9.figshare.10050587.v1

[ref-25] KNBS, Kenya National Bureau of Statistics: Kenya 2009 population and housing census. Vol II: Population and household distribution by socio-economic characteristics. Kenya National Bureau of Statistics, Government of Kenya.2010 Reference Source

[ref-26] KNBS, Kenya National Bureau of Statistics: County Government of Marsabit County Integrated Development Plan, 2013-2017. 2013;16–18. Reference Source

[ref-27] Lamuka PO Njeruh FM Gitao GC: Camel health management and pastoralists’ knowledge and information on zoonoses and food safety risks in Isiolo County, Kenya. *Pastoralism.* 2017;7(1):20 10.1186/s13570-017-0095-z

[ref-28] Mbaya AW Ibrahim UI Apagu ST: Trypanosomosis of The Dromedary Camel ( *Camelus dromedarius*) and its Vectors in The Tsetse-free Arid Zone of North Eastern Nigeria. *Niger Vet J.* 2006;31(3):195–200. 10.4314/nvj.v31i3.68975

[ref-29] Mochabo KO Kitala PM Gathura PB: Community perceptions of important camel diseases in Lapur Division of Turkana District, Kenya. *Trop Anim Health Prod.* 2005;37(3):187–204. 10.1023/b:trop.0000049301.15826.78 15747856

[ref-30] Moore N Messina J: A landscape and climate data logistic model of tsetse distribution in Kenya. *PLoS One.* 2010;5(7):e11809. 10.1371/journal.pone.0011809 20676406 PMC2910741

[ref-31] Mossaad E Salim B Suganuma K: *Trypanosoma vivax* is the second leading cause of camel trypanosomosis in Sudan after *Trypanosoma evansi*. *Parasit Vectors.* 2017;10(1):176. 10.1186/s13071-017-2117-5 28403897 PMC5390396

[ref-32] Mwamuye MM Kariuki E Omondi D: Novel *Rickettsia* and emergent tick-borne pathogens: A molecular survey of ticks and tick-borne pathogens in Shimba Hills National Reserve, Kenya. *Ticks Tick Borne Dis.* 2017;8(2):208–218. 10.1016/j.ttbdis.2016.09.002 28011185

[ref-33] Nelson GS: *Dipetalonema dracunculoides* (Cobbold 1870), from the dog in Kenya: with a note on its development in the louse-fly, *Hippobosca longipennis*. *J Helmint.* 1963;37(3):235–240. 10.1017/S0022149X00003825

[ref-34] Ngaira JM Njagi EN Ngeranwa JJ: PCR amplification of RoTat 1.2 VSG gene in *Trypanosoma evansi* isolates in Kenya. *Vet Parasitol.* 2004;120(1–2):23–33. 10.1016/j.vetpar.2003.12.007 15019140

[ref-35] Njiru ZK Constantine CC Ndung'u JM: Detection of *Trypanosoma evansi* in camels using PCR and CATT/T. * evansi* tests in Kenya. *Vet Parasitol.* 2004;124(3–4):187–199. 10.1016/j.vetpar.2004.06.029 15381299

[ref-36] Njiru ZK Constantine CC Guya S: The use of ITS1 rDNA PCR in detecting pathogenic African trypanosomes. *Parasitol Res.* 2005;95(3):186–192. 10.1007/s00436-004-1267-5 15619129

[ref-37] Oryan A Valinezhad A Bahrami S: Prevalence and pathology of camel filariasis in Iran. *Parasitol Res.* 2008;103(5):1125–1131. 10.1007/s00436-008-1104-3 18629537

[ref-38] Parola P Roux V Camicas JL: Detection of *ehrlichiae* in African ticks by polymerase chain reaction. *Trans R Soc Trop Med Hyg.* 2000;94(6):707–708. 10.1016/S0035-9203(00)90243-8 11198664

[ref-39] Petersen FT Meier R Kutty SN: The phylogeny and evolution of host choice in the Hippoboscoidea (Diptera) as reconstructed using four molecular markers. *Mol Phylogenet Evol.* 2007;45(1):111–122. 10.1016/j.ympev.2007.04.023 17583536

[ref-40] Potgieter FT Sutherland B Biggs HC: Attempts to transmit *Anaplasma marginale* with *Hippobosca rufipes* and *Stomoxys calcitrans*. *Onderstepoort J Vet Res.* 1981;48(2):119–122. 7312304

[ref-41] Probert WS Schrader KN Khuong NY: Real-time multiplex PCR assay for detection of *Brucella* spp., B. *abortus*, and B. *melitensis*. *J Clin Microbiol.* 2004;42(3):1290–1293. 10.1128/jcm.42.3.1290-1293.2004 15004098 PMC356861

[ref-42] Rahola N Goodman SM Robert V: The Hippoboscidae (Insecta: Diptera) from Madagascar, with new records from the "Parc National de Midongy Befotaka". *Parasite.* 2011;18(2):127–40. 10.1051/parasite/2011182127 21678788 PMC3671411

[ref-43] Rani PA Coleman GT Irwin PJ: *Hippobosca longipennis*--a potential intermediate host of a species of *Acanthocheilonema* in dogs in northern India. *Parasit Vectors.* 2011;4:143. 10.1186/1756-3305-4-143 21781294 PMC3161949

[ref-44] Reysenbach AL Giver LJ Wickham GS: Differential amplification of rRNA genes by polymerase chain reaction. *Appl Environ Microbiol.* 1992;58(10):3417–8. 1280061 10.1128/aem.58.10.3417-3418.1992 PMC183115

[ref-45] Scoles GA Broce AB Lysyk TJ: Relative efficiency of biological transmission of *Anaplasma marginale* (Rickettsiales: *Anaplasmataceae*) by Dermacentor andersoni (Acari: Ixodidae) compared with mechanical transmission by *Stomoxys calcitrans* (Diptera: Muscidae). *J Med Entomol.* 2005;42:668–75. 16119558 10.1603/0022-2585(2005)042[0668:REOBTO]2.0.CO;2

[ref-46] Šimenc J Potočnik U: Rapid differentiation of bacterial species by high resolution melting curve analysis. *Appl Biochem Micro+.* 2011;47(3):256–263. 10.1134/S0003683811030136 21790027

[ref-47] SSFR, Smart Survey Final Report: Laisamis and North Horr Survey Zones, Marsabit County 18 ^th^– 29 ^th^January 2017. 2017. Reference Source

[ref-48] Tokarz R Kapoor V Samuel JE: Detection of tick-borne pathogens by MassTag polymerase chain reaction. *Vector Borne Zoonotic Dis.* 2009;9(2):147–52. 10.1089/vbz.2008.0088 18800864 PMC2976645

[ref-49] Truc P Büscher P Cuny G: Atypical human infections by animal trypanosomes. *PLoS Negl Trop Dis.* 2013;7(9):e2256. 10.1371/journal.pntd.0002256 24069464 PMC3772015

[ref-50] Urakawa T Verloo D Moens L: *Trypanosoma evansi*: cloning and expression in *Spodoptera frugiperda* [correction of fugiperda] insect cells of the diagnostic antigen RoTat1.2. *Exp Parasitol.* 2001;99(4):181–9. 10.1006/expr.2001.4670 11888244

[ref-51] Vanhollebeke B Truc P Poelvoorde P: Human *Trypanosoma evansi* infection linked to a lack of apolipoprotein L-I. *N Engl J Med.* 2006;355(26):2752–2756. 10.1056/NEJMoa063265 17192540

[ref-52] Younan M Abdurahman O: Milk and Meat from the Camel: Handbook on Products and Processing.Z. Farah A. Fischer (Editors), vdf Hochschulverlag AG, ETH Zuerich, Zuerich/Singen, Switzerland.2014;67–76. ISBN 372812527X. Reference Source

[ref-53] Young KE Franklin AB Ward JP: Infestation of northern spotted owls by hippoboscid (Diptera) flies in northwestern California. *J Wildl Dis.* 1993;29(2):278–83. 10.7589/0090-3558-29.2.278 8487378

