# Supplementary material for: Detection of blood pathogens in camels and their associated ectoparasitic camel biting keds, Hippobosca camelina: the potential application of keds in xenodiagnosis of camel haemopathogens
Source: AAS Open Res. 2020 May 20;2:164. Originally published 2019 Nov 5. [Version 2] doi: 10.12688/aasopenres.13021.2 (PMC7243205; doi:10.12688/aasopenres.13021.2)
Supplement: Peer review history

# Peer review: Reviewer response for version 2

**DOI:** 10.21956/aasopenres.14165.r27470

**Published:** 2020-05-21

**Authors:** Dennis Muhanguzi

**Affiliations:** 1 School of Biosecurity, Biotechnical and Laboratory Science, College of Veterinary Medicine Animal Resources and Biosecurity, Makerere University, Kampala, Uganda

The authors have implemented all suggestions I made to Version 1 to my satisfaction. Where they were not able to implement the suggested changes, they provided satisfactory explanations or included these challenges as limitations to the study.

Is the work clearly and accurately presented and does it cite the current literature?

Partly

If applicable, is the statistical analysis and its interpretation appropriate?

Not applicable

Are all the source data underlying the results available to ensure full reproducibility?

Yes

Is the study design appropriate and is the work technically sound?

Partly

Are the conclusions drawn adequately supported by the results?

Partly

Are sufficient details of methods and analysis provided to allow replication by others?

Yes

Reviewer Expertise:

Molecular epidemiology, Spatial epidemiology, Veterinary Sciences

I confirm that I have read this submission and believe that I have an appropriate level of expertise to confirm that it is of an acceptable scientific standard.

# Peer review: Reviewer response for version 2

**DOI:** 10.21956/aasopenres.14165.r27469

**Published:** 2020-05-21

**Authors:** Elizabeth A. Opiyo

**Affiliations:** 1 Department of Biology, Faculty of Science, Gulu University, Gulu, Uganda

No further comments.

Is the work clearly and accurately presented and does it cite the current literature?

Yes

If applicable, is the statistical analysis and its interpretation appropriate?

I cannot comment. A qualified statistician is required.

Are all the source data underlying the results available to ensure full reproducibility?

Yes

Is the study design appropriate and is the work technically sound?

Partly

Are the conclusions drawn adequately supported by the results?

Yes

Are sufficient details of methods and analysis provided to allow replication by others?

Yes

Reviewer Expertise:

Vector biology and parasitology

I confirm that I have read this submission and believe that I have an appropriate level of expertise to confirm that it is of an acceptable scientific standard.

# Peer review: Reviewer response for version 1

**DOI:** 10.21956/aasopenres.14109.r27252

**Published:** 2019-11-20

**Authors:** Elizabeth A. Opiyo

**Affiliations:** 1 Department of Biology, Faculty of Science, Gulu University, Gulu, Uganda

*Hippobosca camelina,* also known as Keds sampled from camels and blood samples from the camels from which the Keds were samples, were examined for the presence of pathogens with a view of using Keds for xenodiagnosis of camel haemopathogens. The study took place in Laisamis, northern Kenya. The samples were screened using PCR and PCR-HRM and revealed the presence of epizootic pathogens in camels from Kenya and suggests that Keds have potential use in diagnosis of heamopathogens circulating in camels.

 In the study design and sampling, no particular sample size or criteria appear to have been planned in advance. If this was opportunistic sampling then this should be stated and justified.

 Regarding molecular diagnosis of pathogens, conventional PCR was used for detection of *C. burnetii, Anaplasma* spp, *Ehrlichia* spp, *Brucella* spp and for piroplasma belonging to *Theileria* and *Babesia* PCR-HRM and for trypanosomes conventional PCR was followed by visualization and later gel electrophoresis and sequencing. The lack of uniformity in the analysis of the pathogens requires some explanation/justification for the readers that may be interested in doing the same analysis for their studies.

Is the work clearly and accurately presented and does it cite the current literature?

Yes

If applicable, is the statistical analysis and its interpretation appropriate?

I cannot comment. A qualified statistician is required.

Are all the source data underlying the results available to ensure full reproducibility?

Yes

Is the study design appropriate and is the work technically sound?

Partly

Are the conclusions drawn adequately supported by the results?

Yes

Are sufficient details of methods and analysis provided to allow replication by others?

Yes

Reviewer Expertise:

Vector biology and parasitology

I confirm that I have read this submission and believe that I have an appropriate level of expertise to confirm that it is of an acceptable scientific standard.

# Response

**Published:** 2020-05-07

**Authors:** Joel L. Bargul

**Affiliations:** International Centre of Insect Physiology and Ecology, Kenya

**Reviewer: **In the study design and sampling, no particular sample size or criteria appear to have been planned in advance. If this was opportunistic sampling then this should be stated and justified.

**Response: **The type of sampling is now specified as opportunistic sampling and was adopted for convenience by sampling camels from diverse geographical locations as they converge at specific water drinking points. Otherwise it will be challenging to conduct daily sampling of camels considering that camel owners are nomadic pastoralists with busy lifestyles characterized by long distance movements together with their animals and other belongings.

**Reviewer: **Regarding molecular diagnosis of pathogens, conventional PCR was used for detection of *C. burnetii*, *Anaplasma* spp, *Ehrlichia* spp, *Brucella* spp and for piroplasma belonging to *Theileria* and *Babesia* PCR-HRM and for trypanosomes conventional PCR was followed by visualization and later gel electrophoresis and sequencing. The lack of uniformity in the analysis of the pathogens requires some explanation/justification for the readers that may be interested in doing the same analysis for their studies.

**Response:** PCR-HRM assays (i.e. combination of both conventional PCR and HRM) were used in detection of all listed pathogens above, except trypanosomes whose detection protocols by HRM are not established at present hence sufficiently screened using conventional ITS-1 PCR by Njiru et al (2005) followed by gene sequencing. The advantage of HRM is that helps in selection of representative samples producing unique HRM melting curves for gene sequencing thus saving on cost.

# Peer review: Reviewer response for version 1

**DOI:** 10.21956/aasopenres.14109.r27253

**Published:** 2019-11-18

**Authors:** Dennis Muhanguzi

**Affiliations:** 1 School of Biosecurity, Biotechnical and Laboratory Science, College of Veterinary Medicine Animal Resources and Biosecurity, Makerere University, Kampala, Uganda

**Reviewer’s Summary**

 Kidambasi K.O *et al.* set out to identify the most important Arthropod-Vectored *C. dromedarius *pathogens and those in *Hippobosca* *camelina* [Camel Keds] as well as to asses the potential use of camel keds in xenodiagnosis of *C. dromedarius haemo*pathogens in Marsabit county, Northern Kenya. They used a battery of molecular techniques to identify the most important parasites circulating in camels [n=249] and Camel Keds [n=117] as *T. vivax and* *T. evansi.* As well, *Candidatus A. camelli *was detected in camels and not Camel keds while *T. melophagium* was detected in Camel keds and not Camels. Entomological techniques were used to identify *Stomoxys calcitrans* as the major biting fly in this region. Given a close match of pathogens detected in camels and camel keds that were sampled from them, the authors  discuss herein the potential use of camel keds in xenodiagnosis of camel haemopathogens and the animal and public health roles of the identified hemopathogens.

 This is a good manuscript in its area but needs major changes to further improve its quality and scientific merit.

**Minor Changes [discretionary]**

 Please consider implementing the following minor changes

1. Throughout this manuscript, change the phrase….”disease pathogens” to ‘pathogens’ because all pathogens cause disease
2. Please consider removing absolute fractions in the results section of the abstract given that you made mention of the number of samples analysed in the methods section of this abstract. If you decide to maintain them, please write them as; Trypanosoma vivax; 41 % [102/249], Trypanosoma evansi; 1.2 % [3/249]. You have indicated that only 200 camel blood samples were analysed to arrive at the prevalence of *Candidatus A. camelli*. I was unable to find a reason for this in the methods and materials section. Please cross check that this was not quoted in error. In case you did analyze 200 instead of 249 camel blood samples please explain this choice in your materials and methods section.
3. Please consider changing ---unpredictable rainfalls to … unpredictable rainfall
4. Rephrase the first sentence of the third paragraph of this section. You can as well break this sentence into two sentences i.e. Hippoboscids (keds) are obligate hematophagous ectoparasites of mammals and birds. They belong to the family Hippoboscidae within the superfamily Hippoboscoidae (Petersen et al., 2007 ^1^; Rahola et al., 2011 ^2^).

**Methods and materials**

1. **Weather conditions**: ' ….short and long rains to'……short and long wet seasons
2. **Study design and sample collection**: '……samples were collected after receiving informed verbal consent from camel keepers. All camel keepers were neither able to read nor write, thus verbal rather than the written consent was adopted as the pragmatic approach…' This sentence should be moved to Ethical approval on page 4 of 11.
3. **Ethical approval [page 4 of 11]**: please delete the sentence that begins with JB…the principal investigator…
4. **DNA extraction [Page 4 of 11]**: unless it is the journal requirement, 2.0-mm or 1.5-mL; change this to 2.0 mm or 1.5 mL
5. **Detection of pathogen DNA [page 5 of 11]:** Anaplasma and Ehrlichia were detected by PCR amplification using genus-specific primers…. should be changed to …. Anaplasma and Ehrlichia species were detected by PCR amplification using genus-specific primers. Please rewrite the sentence……….. Brucella arbutus forward primer, B. melitensis forward primer, and Brucella spp. universal reverse primer targeting the IS711 gene (Probert et al., 2004), 5.5 μL PCR water and 1.0 μL of template DNA. …..Camel pox…. in the last paragraph of this section as well as table 1 should be changed to Camel pox Virus [CMLV]. As you will remember, you did not detect the disease but CMLV genetic material.
6. **DNA purification and sequencing [page 5 of 11].** Second sentence of this section …..The PCR amplicons….is incomplete. Please complete this sentence.
7. **Data analysis**: Please delete the sentence that begins with….. Ground truthing….Ground truthing applies more to remote sensing and machine learning. You just need to explain how the map in figure 1 was drawn in the sentence that follows. Here you will need to mention the ArcGIS v. 10.6 extension that you used to complete this map.
8. **Results:** Please transfer contents of paragraph 4 that start with ….sequences obtained in the study… to an appropriate section under methods and materials. As well, explanatory text of Table 3 on page 8 of 11 sounds like methods and materials information. Please keep that in methods and materials and provide a stand-alone legend for this table if required.
9. ** Major comments: Methods and Materials**: There is need to include sections on Sample size determination and sampling strategy as well as to improve the current sub-sections under this section. In your introduction section, you indicated that about 3 million camels are kept in northern Kenya. Under methods and materials, there is no explanation of how many of these 3 million camels are kept in Laisamis or even Marsabit County. Reading this manuscript the following questions arise. Are 249 camels sampled over 5 days period  representative of **n** camels in the study country or Laisamis zone? Are the Laisamis camels representative of all the 3 million camels in Nothern Kenya? Were all the camels presented in the 5 sampling days sampled so long as their owners consented to the study?, If not, how were the 249 camels sampled from **n _2_** camels presented during the 5 sampling days? Why was sampling only done in September [short wet season]?  How were the 21 sampled herds [at 4 sites] arrived at? How many herds were there in the county and how were the 21 herds selected from all the county camel herds? What is the definition of a herd given that animals that are owned in a communal pastoral husbandry obtaining mix-up? What was the sampling unit? How were the sites for biting fly trapping selected and what was the inter trap distance? etc…\
10. Data analysis needs to be revisited. The fly apparent density in table 3 can be well presented spatially. To be able to discuss possibility of mechanical transmission of different hemopathogens by different biting flies e.g. Stomoxys the association between fly apparent density and hemopathogen prevalence needs to be adjusted for potential spatial dependence. This you can do using generalized least squares model with a Gaussian spatial correlation structure to quantify the effect or other appropriate models.
11. Please include a pairwise alignment of *Candidatus A. camelli* from this study and those from the GenBank as was done for *T. melophagium*
12. **Discussion: **I would like to draw the authors’ attention to some of the following discussion sections which I strongly believe they need to revisit.
13. First paragraph [Page 7 of 11]: Associating the high prevalence of hemopathogens in camels to the potential of camel keds in hemopathogen transmission is not supported by the results of this study. Note that keds were only less than 2% of all biting flies trapped. This can only be attributed to mechanical transmission of these hemopathogens by *Stomoxys Calicitrans* and Tabanids which were >98% of all biting flies trapped. You can be authoritative about these associations if you improve data analysis as recommended in comment II above
14. Pursuant to comment 13 above, you need to rewrite the hypothesis you make at the end of paragraph 1 of discussion section on page 7 of 11. If Mechanical transmission of camel hemopathogens were important in this region, it would rather be by Stomoxys and other Tabanids and not camel keds [camel keds were <2% of all biting flies trapped]; moreover you did not rule out or in potential spatial dependence between hemoparasite prevalence and  fly apparent density.
15. Paragraph 2 of discussion [page 7 of 11]. There is mention of reports that support absence of tsetse flies in the study area and yet no references of such reports are included. When I checked this fact myself, I found that this study area has recently been cited as an area with high risk of tsetse infestation using robust landscape and climatic data modeling ^3^.
16. Last paragraph on page 7 of 11; The variations in the micro-climatic conditions, differences in the study designs and time lapse are the most likely explanations for the differences in *T. evansi* prevalence in camels previously reported in other parts of Kenya and in this study.
17. There is need to include a discussion of the limitations of this study [see major comment I & VII above. Think of snap short sampling during short wet season? seasonality vs hemoparasite and vector density etc
18. There is need to nuance the recommendation about heightening public and veterinary surveillance of *T. evansi *as a zoonotic hemoparasite [first paragraph, page 9 of 11] because there are no reported major outbreaks of this atypical human African trypanosomiasis either in Kenya or elsewhere ever reported. The only cases of atypical *T. evansi* human infections have been reported in either immunocompromised or accidental infections ^4^ that do not warrant setting up veterinary and public health surveillance programs.
19. Second last sentence; paragraph 2, page 9 of 11….it is conceivable …. . This needs to be reinterpreted. Finding *Candidatus A. cameli *in keds and camels certainly means that keds are feeding predominantly on camels positive for *Candidatus A. cameli. This study design was *not to prove mechanical transmission of *Candidatus A. cameli *by Keds; given that previous mechanical transmission studies were not able to prove that, it is not conceivable in a study of such a design to make this assertion.
20. Second sentence of paragraph 4 page 9 of 11…….detection of similar haemopathogens in these camel flies…… This needs to be reinterpreted. Finding a similar repertoire of hemoparasites in keds and in camels only indicates that keds were feeding on hemoparasite positive camels. Only 2 % of the caught biting flies were keds and you can’t emphasize ked mechanical transmission than that of Tabanids and Stomoxys which were > 98% of all biting flies trapped; with known mechanical transmission potential. The only application you can make out of this result is about xenodiagnosis and not mechanical transmission of hemoparasites by keds unless this is proven in study with suitable study design or you can refer to literature!
21. Page 10 of 11, first sentence: The point you make about xenodiagnosis saving time and money needs to be substantiated. It takes as much time to collect keds from camels as it takes to take blood samples from camels. If similar diagnostic methods are used to detect pathogens in keds and camels, I would not anticipate pathogen detection in keds to be any cheaper than pathogen detection in camels? can you please discuss how xenodiagnosis would be cheaper and shorter than detection of pathogens directly from camel blood?
22. Last paragraph of discussion section, page 10 of 11. ; …..Molecular detection of *T. melophagium*….

Note that detection of this parasite in a ked does not mean that such a ked was infected with *T. melophagium*. Unless proven, it would mean that it had consumed a blood meal from a host [might not be camel at all since no camel was found positive for genetic material of this parasite] that had been positive for *T. melophagium* genetic material. This has nothing to do with *H. camelina* being able to transmit [biologically or mechanically] *T. melophagium.*

**Conclusion**

 This needs to be refined after refining the discussion. Blood samples need not to be taken from the Jugular. You can these days take blood samples [125 ul]  from ear veins and have them preserved on FTA cards. Unless substantiated as in comment XII, this conclusion has to be rewritten so that it is supported by the findings of this study.  

**References**

 As indicated in my comments above, the attention of the authors is drawn to some of the key literature they were not able to refer to in their discussion section e.g Truc *et al*. ^4^, Moore *et al*. ^3^.

Is the work clearly and accurately presented and does it cite the current literature?

Partly

If applicable, is the statistical analysis and its interpretation appropriate?

Not applicable

Are all the source data underlying the results available to ensure full reproducibility?

Yes

Is the study design appropriate and is the work technically sound?

Partly

Are the conclusions drawn adequately supported by the results?

Partly

Are sufficient details of methods and analysis provided to allow replication by others?

Yes

Reviewer Expertise:

Molecular epidemiology, Spatial epidemiology, Veterinary Sciences

I confirm that I have read this submission and believe that I have an appropriate level of expertise to confirm that it is of an acceptable scientific standard, however I have significant reservations, as outlined above.

## References

[1] : Protective effect of antibiotics against serious complications of common respiratory tract infections: retrospective cohort study with the UK General Practice Research Database. *BMJ*.2007;335(7627) : 10.1136/bmj.39345.405243.BE 982 10.1136/bmj.39345.405243.BE 17947744PMC2072032

[2] : The Hippoboscidae (Insecta: Diptera) from Madagascar, with new records from the "Parc National de Midongy Befotaka". *Parasite*.2011;18(2) : 10.1051/parasite/2011182127 127-40 10.1051/parasite/2011182127 21678788PMC3671411

[3] : A landscape and climate data logistic model of tsetse distribution in Kenya. *PLoS One*.2010;5(7) : 10.1371/journal.pone.0011809 e11809 10.1371/journal.pone.0011809 20676406PMC2910741

[4] : Atypical human infections by animal trypanosomes. *PLoS Negl Trop Dis*.2013;7(9) : 10.1371/journal.pntd.0002256 e2256 10.1371/journal.pntd.0002256 24069464PMC3772015

# Response

**Published:** 2020-05-07

**Authors:** Joel L. Bargul

**Affiliations:** International Centre of Insect Physiology and Ecology, Kenya

**Minor Changes [discretionary]**

Please consider implementing the following minor changes

1.     Throughout this manuscript, change the phrase….”disease pathogens” to ‘pathogens’ because all pathogens cause disease

**Response:** This has been corrected throughout the manuscript.

2.     Please consider removing absolute fractions in the results section of the abstract given that you made mention of the number of samples analysed in the methods section of this abstract. If you decide to maintain them, please write them as; *Trypanosoma vivax*; 41 % [102/249], *Trypanosoma evansi*; 1.2 % [3/249]. You have indicated that only 200 camel blood samples were analysed to arrive at the prevalence of “ *Candidatus* A. camelii”. I was unable to find a reason for this in the methods and materials section. Please cross check that this was not quoted in error. In case you did analyze 200 instead of 249 camel blood samples please explain this choice in your materials and methods section.

**Response 1: **absolute fractions have been removed from the results section of the Abstract.

**Response 2:** we analyzed a total of 249 samples as specified under ‘Materials and Methods’ section, and not 200 as quoted in the Abstract. We apologize for this error that is now corrected.

3.     Please consider changing ---unpredictable rainfalls to … unpredictable rainfall

**Response:** done.

4.     Rephrase the first sentence of the third paragraph of this section. You can as well break this sentence into two sentences i.e. Hippoboscids (keds) are obligate hematophagous ectoparasites of mammals and birds. They belong to the family Hippoboscidae within the superfamily Hippoboscoidae (Petersen et al., 20071; Rahola et al., 20112).

**Response: **done.

Methods and materials

5.     Weather conditions: ' ….short and long rains to'……short and long wet seasons

**Response:** Done

6.     Study design and sample collection: '……samples were collected after receiving informed verbal consent from camel keepers. All camel keepers were neither able to read nor write, thus verbal rather than the written consent was adopted as the pragmatic approach…' This sentence should be moved to Ethical approval on page 4 of 11.

**Response:** Above sentences have now been moved to “Ethical approval” section.

7.     Ethical approval [page 4 of 11]: please delete the sentence that begins with JB…the principal investigator…

**Response: **Deleted

8.     DNA extraction [Page 4 of 11]: unless it is the journal requirement, 2.0-mm or 1.5-mL; change this to 2.0 mm or 1.5 mL

**Response:** Done

9.     Detection of pathogen DNA [page 5 of 11]: *Anaplasma* and *Ehrlichia* were detected by PCR amplification using genus-specific primers…. should be changed to …. *Anaplasma* and *Ehrlichia* species were detected by PCR amplification using genus-specific primers.

**Response:** Done

Please rewrite the sentence……….. *Brucella* *arbutus* forward primer, *B. melitensis* forward primer, and *Brucella *spp. universal reverse primer targeting the IS711 gene (Probert et al., 2004), 5.5 μL PCR water and 1.0 μL of template DNA. …..Camel pox…. in the last paragraph of this section as well as table 1 should be changed to Camel pox Virus [CMLV]. As you will remember, you did not detect the disease but CMLV genetic material.

**Response:** Rephrased

10.  DNA purification and sequencing [page 5 of 11]. Second sentence of this section …..The PCR amplicons….is incomplete. Please complete this sentence.

**Response:** we apologize for this mistake and thank you for being very keen; the word ‘were’ is now inserted to complete the sentence.

11.  Data analysis: Please delete the sentence that begins with….. Ground truthing….Ground truthing applies more to remote sensing and machine learning. You just need to explain how the map in figure 1 was drawn in the sentence that follows. Here you will need to mention the ArcGIS v. 10.6 extension that you used to complete this map.

**Response:** We thank the Reviewer for this suggestion that we have effected to improve the manuscript.

12.  Results: Please transfer contents of paragraph 4 that start with ….sequences obtained in the study… to an appropriate section under methods and materials.

**Response:** this paragraph is now appropriately placed under ‘methods and materials’ section.

As well, explanatory text of Table 3 on page 8 of 11 sounds like methods and materials information. Please keep that in methods and materials and provide a stand-alone legend for this table if required.

**Response:** Many thanks for this comment, we have re-written heading of Table 3.

13.  Major comments: Methods and Materials: There is need to include sections on Sample size determination and sampling strategy as well as to improve the current sub-sections under this section. In your introduction section, you indicated that about 3 million camels are kept in northern Kenya. Under methods and materials, there is no explanation of how many of these 3 million camels are kept in Laisamis or even Marsabit County.

Reading this manuscript the following questions arise. Are 249 camels sampled over 5 days period representative of n camels in the study country or Laisamis zone? Are the Laisamis camels representative of all the 3 million camels in Nothern Kenya? Were all the camels presented in the 5 sampling days sampled so long as their owners consented to the study?, If not, how were the 249 camels sampled from n2 camels presented during the 5 sampling days? Why was sampling only done in September [short wet season]?  How were the 21 sampled herds [at 4 sites] arrived at? How many herds were there in the county and how were the 21 herds selected from all the county camel herds? What is the definition of a herd given that animals that are owned in a communal pastoral husbandry obtaining mix-up? What was the sampling unit? How were the sites for biting fly trapping selected and what was the inter trap distance? etc…

**Response:** more information is now provided, for instance, a recent study reported a total population of 203,320 camels in Marsabit County, where our present study was conducted (SSFR, 2017).

This field study was cross-sectional in design and involved opportunistic sampling, whereby we sampled camels converging at the water drinking points. This type of sampling was most convenient due to the nomadic pastoralist lifestyle involving frequent long distance movements. We sampled all camels in the herds found at water drinking points for 5 consecutive days in the dry season (September 2017). Due lack of data, we could not calculate the sample sizes, thus we collected as many samples as possible during the sampling duration.

Sampling was preferred in dry season of September because then the camel ked densities are high, unlike during wet season.

Flies were randomly collected from 21 camel herds (that was possible in 5 days) and we targeted to collect as many keds infesting camels as possible. The herds were from four sites. We do not have data on the number of herds in this community whose main occupation of the Household Heads is livestock herding at 87%, followed by Casual Labor (SSFR, 2017). We defined a camel herd as one under care of a specific farmer and it comprises of camels that graze and stay together most of the time. Much as we tried to avoid sampling of camel herds that mostly co-graze, this did not affect our objective of studying pathogens in camels and keds kept under natural setting. All camels, in each herd that ranged from 8 – 90 camels, were sampled and we aimed to collect all camel keds from the sampled camel herds.

The sites for biting fly trapping were selected near livestock pens and next to watering points along Laisamis and Koya Rivers. The inter trap distance was at least 100 meters.

14.  Data analysis needs to be revisited. The fly apparent density in Table 3 can be well presented spatially. To be able to discuss possibility of mechanical transmission of different haemopathogens by different biting flies e.g. *Stomoxys* the association between fly apparent density and hemopathogen prevalence needs to be adjusted for potential spatial dependence. This you can do using generalized least squares model with a Gaussian spatial correlation structure to quantify the effect or other appropriate models.

**Response:** It is not possible to do these analyses with our limited data. Our key focus was on camel keds infesting camels. Since our preliminary data showed high prevalence of camel trypanosomiasis in the study region, we therefore wanted to check whether tsetse flies are present to cause disease transmissions. Three traps were deployed per site on daily basis. Daily trap collections were pooled, fly species sorted, counted, and preserved in absolute ethanol ready for transportation to the Nairobi-based laboratories at icipe (Table 3).

15.  Please include a pairwise alignment of “ *Candidatus* A. camelii” from this study and those from the GenBank as was done for *T. melophagium*

**Response:** we thank the reviewer for this helpful addition. This alignment is now provided as Figure 5.

16.  Discussion: I would like to draw the authors’ attention to some of the following discussion sections, which I strongly believe they need to revisit.

17.  First paragraph [Page 7 of 11]: Associating the high prevalence of hemopathogens in camels to the potential of camel keds in hemopathogen transmission is not supported by the results of this study. Note that keds were only less than 2% of all biting flies trapped. This can only be attributed to mechanical transmission of these hemopathogens by *Stomoxys Calcitrans* and Tabanids which were >98% of all biting flies trapped. You can be authoritative about these associations if you improve data analysis as recommended in comment II above

**Response:** As described under ‘materials and methods’ and ‘discussion’ sections, camel keds do not normally leave their host (unless when disturbed) as they firmly attach to the hairs on the camel’s skin by their tarsal claws during feeding or resting. During our study, keds were common on the camels which was not the case for other biting flies. The keds can hop from one camel to another when disturbed and if they are contaminated they could transmit the pathogens to the next host as shown by preliminary findings of our ongoing studies (Bargul et al., unpublished). Thus, our intention to deploy fly traps was mainly to catch tsetse flies and other biting fly species but not keds as we understand at present that efficient traps for keds are not available and the monoconical traps we deployed are efficient at trapping tsetse flies, as well as biting flies such as *Stomoxys* and Tabanids, with house flies often being non-targets. Our ongoing studies aim at designing ked-specific traps. It is very likely that the few trapped keds comprising of 0 - 2% of total biting fly catches were off targets.

18.  Pursuant to comment 13 above, you need to rewrite the hypothesis you make at the end of paragraph 1 of discussion section on page 7 of 11. If Mechanical transmission of camel haemopathogens were important in this region, it would rather be by *Stomoxys* and other Tabanids and not camel keds [camel keds were <2% of all biting flies trapped]; moreover you did not rule out or in potential spatial dependence between haemoparasite prevalence and fly apparent density.

**Response: **please refer to #13 above that partially addresses this question.

Although *Stomoxys* and Tabanids are potential mechanical vectors of pathogens as previously reported, we do not have data to affirm their vectorial competence in disease transmission among camels in northern Kenya. The major focus of our study was on the ectoparasitic camel keds, but not on the other biting flies that were often absent on camels, unlike keds. In fact, our motivation to deploy traps was to determine occurrence of tsetse flies (definitive biological vectors of African trypanosomes) as camel trypanosomiasis was detected in almost half of the sampled camels. Our preliminary findings from ongoing studies show evidence of *Anaplasma* transmission by camel keds from naturally infected dromedary camels to laboratory-reared mice and rabbits (Bargul et al., unpublished). We are also testing trypanosome transmission capacity of camel keds as keds are the closest tsetse relatives both belonging to same superfamily.

19.  Paragraph 2 of discussion [page 7 of 11]. There is mention of reports that support absence of tsetse flies in the study area and yet no references of such reports are included. When I checked this fact myself, I found that this study area has recently been cited as an area with high risk of tsetse infestation using robust landscape and climatic data modeling3.

**Response: ** Despite the high risk prediction for tsetse infestation in our study area (Moore and Messina, 2010), we did not collect any tsetse flies during the sampling period. Additionally, during our community and public engagement sessions, the camel farmers reported absence of these flies in Laisamis, but in the far regions such as Meru County, over 200 km away.

20.  Last paragraph on page 7 of 11; The variations in the micro-climatic conditions, differences in the study designs and time lapse are the most likely explanations for the differences in *T. evansi* prevalence in camels previously reported in other parts of Kenya and in this study.

**Response: **these factors that could influence disease prevalence are now better reflected in the discussion, i.e.

“These regional variations in prevalence of *T. evansi* infection could result from seasonal disease outbreaks, variations in the micro-climatic conditions, disease stability in endemic zones, and the presence of competent insect-vectors, among other factors, including differences in the study designs and time lapse. Our study design has key limitations in comparative studies because sampling was not done during wet season, thus the relationship between seasonality versus prevalence of haemoparasites and vector density could not be established”

21.  There is need to include a discussion of the limitations of this study [see major comment I & VII above. Think of snapshot sampling during short wet season? Seasonality vs haemoparasite and vector density, etc.

**Response:** limitations of our study design are now highlighted in the discussion. Please see #17 above.

22.  There is need to nuance the recommendation about heightening public and veterinary surveillance of *T. evansi* as a zoonotic haemoparasite [first paragraph, page 9 of 11] because there are no reported major outbreaks of this atypical human African trypanosomiasis either in Kenya or elsewhere ever reported. The only cases of atypical *T. evansi* human infections have been reported in either immunocompromised or accidental infections that do not warrant setting up veterinary or public health surveillance programs.

**Response:** we fully agree that *T. evansi* human infections are very uncommon, thus we deleted our earlier suggestion proposing “increased surveillance by veterinary and public health partners to mitigate spread of *T. evansi* in humans”

23.  Second last sentence; paragraph 2, page 9 of 11….it is conceivable …. . This needs to be reinterpreted. Finding “ *Candidatus* A. camelii” in keds and camels certainly means that keds are feeding predominantly on camels positive for “ *Candidatus* A. camelii”. This study design was not to prove mechanical transmission of “ *Candidatus* A. camelii” by Keds; given that previous mechanical transmission studies were not able to prove that, it is not conceivable in a study of such a design to make this assertion.

**Response: **yes, it is true that the focus of this study was not to prove mechanical transmission of pathogens. However, we show that identical *Anaplasma* species in camels and keds collected from them, suggesting that keds fed on *Anaplasma*-positive camels. Based on this finding, we hypothesize that in the process of blood-feeding, keds, just like *Stomoxys calcitrans*, could mechanically transmit anaplasmosis via contaminated mouthparts. We have preliminary data, from another ongoing study, to support “ *Candidatus* A. camelii”-transmission by camel keds from naturally-infected camels to mice and rabbits (Bargul et al., unpublished).

24.  Second sentence of paragraph 4 page 9 of 11…….detection of similar haemopathogens in these camel flies…… This needs to be reinterpreted. Finding a similar repertoire of haemoparasites in keds and in camels only indicates that keds were feeding on haemoparasite positive camels. Only 2% of the caught biting flies were keds and you can’t emphasize ked mechanical transmission than that of Tabanids and *Stomoxys* which were > 98% of all biting flies trapped; with known mechanical transmission potential. The only application you can make out of this result is about xenodiagnosis and not mechanical transmission of haemoparasites by keds unless this is proven in study with suitable study design or you can refer to literature!

**Response: **please note that this is already addressed under response #13, #14, & #19 above.

25.  Page 10 of 11, first sentence: The point you make about xenodiagnosis saving time and money needs to be substantiated. It takes as much time to collect keds from camels as it takes to take blood samples from camels. If similar diagnostic methods are used to detect pathogens in keds and camels, I would not anticipate pathogen detection in keds to be any cheaper than pathogen detection in camels? Can you please discuss how xenodiagnosis would be cheaper and shorter than detection of pathogens directly from camel blood?

**Response:** Collection of keds off camels was much easier and required relatively less time than blood sampling., We employed six field assistants to restrain each camel for blood collection, a veterinary personnel who collected blood samples, and additional three assistants to carry cool boxes and consumables, ensure accurate labeling of samples and storage, and recording of baseline data. On the other hand, only about four field assistants were needed to collect keds from camel herds, resulting in >50% reduction in labour costs and the required human resource. Fly collection also took shorter time as it was not necessary to restrain camels. Importantly, this xenosurveillance detection provides a less invasive approach than the currently available painful blood collection procedures that pose huge risk to the handlers as camels could occasionally cause severe and even fatal injuries through bites (Abu-Zidan *et al.,* 2012) or by kicking with their legs.

26.  Last paragraph of discussion section, page 10 of 11. ; …..Molecular detection of *T. melophagium*….

Note that detection of this parasite in a ked does not mean that such a ked was infected with *T. melophagium*. Unless proven, it would mean that it had consumed a blood meal from a host [might not be camel at all since no camel was found positive for genetic material of this parasite] that had been positive for *T*. *melophagium *genetic material. This has nothing to do with *H. camelina* being able to transmit [biologically or mechanically] *T. melophagium*.

**Response: **we agree with the reviewer, and subsequently this sentence has been re-written to ensure accurate delivery of information.

Conclusion:

This needs to be refined after refining the discussion. Blood samples need not to be taken from the Jugular. You can these days take blood samples [125 ul] from ear veins and have them preserved on FTA cards. Unless substantiated as in comment XII, this conclusion has to be rewritten so that it is supported by the findings of this study. 

**Comment: **with the above clarification on xenodiagnosis under #21, our conclusions are now well supported.

References

As indicated in my comments above, the attention of the authors is drawn to some of the key literature they were not able to refer to in their discussion section e.g Truc et al.4, Moore et al.3.

**Comment:** many thanks to the Reviewer for your helpful suggestions. We have now added the following references; Rahola et al., 2011, Nelson, 1963, Moore and Messina, 2010, Vanhollebeke et al., 2006, Truc et al., 2013

References

1.     Petersen I, Johnson AM, Islam A, Duckworth G, et al.: Protective effect of antibiotics against serious complications of common respiratory tract infections: retrospective cohort study with the UK General Practice Research Database.BMJ. 2007; 335 (7627): 982 PubMed Abstract | Publisher Full Text (not cited as it seems unrelated to our study)

2.     Rahola N, Goodman SM, Robert V: The Hippoboscidae (Insecta: Diptera) from Madagascar, with new records from the "Parc National de Midongy Befotaka".Parasite. 2011; 18 (2): 127-40 PubMed Abstract | Publisher Full Text (was already cited)

3.     Moore N, Messina J: A landscape and climate data logistic model of tsetse distribution in Kenya.PLoS One. 2010; 5 (7): e11809 PubMed Abstract | Publisher Full Text (cited)

4.     Truc P, Büscher P, Cuny G, Gonzatti MI, et al.: Atypical human infections by animal trypanosomes.PLoS Negl Trop Dis. 2013; 7 (9): e2256 PubMed Abstract | Publisher Full Text (was already cited)
